# Patterns of Playing-Related Musculoskeletal Disorders Among Bangladeshi Tabla Players: A Descriptive Cross-Sectional Study

**DOI:** 10.7759/cureus.107680

**Published:** 2026-04-24

**Authors:** Md. Israt Hasan, Syed Mozaffar Ahmed, Md. Ali Emran, Fatema Newaz, Taufiq Morshed, Mohammed Emran

**Affiliations:** 1 Department of Physical Medicine and Rehabilitation, Sher-E-Bangla Medical College, Barishal, BGD; 2 Department of Physical Medicine and Rehabilitation, Bangladesh Medical University, Dhaka, BGD; 3 Department of Physical Medicine and Rehabilitation, Kumudini Women's Medical College and Hospital, Tangail, BGD; 4 Department of Orthopedic Surgery, National Institute of Traumatology and Orthopedic Rehabilitation, Dhaka, BGD; 5 Department of Physical Medicine and Rehabilitation, Khwaja Yunus Ali Medical College, Sirajganj, BGD

**Keywords:** bangladesh, musicians’ health, performing arts medicine, playing-related musculoskeletal disorders, tabla players

## Abstract

Introduction: Playing-related musculoskeletal disorders (PRMDs) are a major occupational concern in the emerging field of performing arts medicine. They are common among instrumental musicians and may significantly impair performance and quality of life. Percussionists, including tabla players, are particularly vulnerable due to repetitive upper limb movements and sustained postures. However, evidence from South Asia, especially Bangladesh, remains limited. Therefore, this study aimed to describe the prevalence, distribution, and characteristics of PRMDs among Bangladeshi tabla players.

Methods: This descriptive cross-sectional study included 116 professional tabla players with at least five years of playing experience, recruited through purposive sampling from music academies and cultural institutions in Bangladesh. Data were collected using a structured, Bengali-translated questionnaire adapted from validated PRMD assessment tools. Information on demographics, playing habits, musculoskeletal symptoms, and preventive practices was obtained. Data were analyzed using descriptive statistics.

Results: Among the participants (n = 116), 72% reported experiencing PRMDs at some point in their lifetime, while 26% reported current symptoms. The upper limb was the most commonly affected region (63%), with the wrist (27%) and shoulder (26%) being the most frequently involved anatomical sites. Lower back (22%) and fingers (20%) were also commonly affected. A majority (62%) experienced involvement of multiple regions. Many participants reported that PRMDs interfered with playing ability (58%) and daily activities (42%). A descriptive trend suggested a higher prevalence of PRMDs among players with longer daily practice duration. Preventive measures were infrequently practiced, with 53% reporting no preventive strategies.

Conclusion: PRMDs are highly prevalent among Bangladeshi tabla players, predominantly affecting the upper limb and impacting both performance and daily functioning. The limited use of preventive strategies highlights the need for increased awareness, ergonomic training, and rehabilitation interventions tailored to musicians.

## Introduction

Bangladesh has a rich cultural heritage with a vibrant tradition of music and performing arts. Despite the large number of musicians, there is limited structured healthcare provision dedicated to the specific needs of performing artists. Globally, performing arts medicine is an emerging multidisciplinary field, and performing artists remain relatively underserved in terms of injury prevention, specialized care, and medical coverage [1,2].

Playing-related musculoskeletal disorders (PRMDs) are increasingly recognized as a significant occupational health concern among musicians [3]. These disorders encompass a range of musculoskeletal conditions resulting from repetitive movements, sustained postures, and performance-related physical demands [4]. PRMDs may adversely affect performance quality, career longevity, and overall well-being, making them a key focus within the emerging field of performing arts medicine [5].

Percussionists represent a unique subgroup of musicians due to the dynamic and repetitive striking movements required during performance [6]. Tabla players, in particular, typically perform in a seated posture with forward inclination, involving extensive and coordinated use of the upper limbs, wrists, and fingers [7]. These biomechanical demands may predispose them to specific patterns of musculoskeletal disorders [8].

While PRMDs have been extensively studied among Western orchestral musicians [9], evidence from South Asia particularly among traditional instrumentalists such as tabla players remains limited [7]. This gap highlights the need for context-specific research to better understand risk patterns and inform preventive strategies.

Therefore, this study aimed to assess the prevalence and anatomical distribution of PRMDs among Bangladeshi tabla players (primary objective), and to describe associated characteristics, including symptom severity, functional impact, and preventive practices (secondary objectives).

## Materials and methods

Study design and participants

This descriptive cross-sectional study was conducted among professional tabla players in Bangladesh over a one-year period from July 2023 to June 2024. A total of 116 participants with at least five years of playing experience were included using purposive sampling from music academies, cultural organizations, and performance venues. Data were collected through in-person administration of the questionnaire at these settings. A non-probability purposive (convenience) sampling approach was used due to the limited accessibility of professional tabla players within the study setting.

Inclusion and exclusion criteria

Professional tabla players with a minimum of five years of playing experience were included. Individuals with a history of trauma, congenital musculoskeletal or neurological conditions, or unwillingness to participate were excluded.

Sample size and sampling technique

The required sample size was calculated using the formula for finite population described by Krejcie and Morgan [10]:

S = X²NP(1−P) / [d²(N−1) + X²P(1−P)]

where S = required sample size, N = population size, P = estimated prevalence, d = margin of error, and X² = chi-square value at 95% confidence level (3.841).

The population of professional tabla players was estimated to be approximately 700. The expected prevalence (P) was assumed to be 13% based on prior literature, with a margin of error (d) of 5%. The calculated sample size was approximately 174 participants. However, due to practical constraints including limited availability of eligible participants and time limitations during the study period, a total of 116 participants were included using a convenience sampling technique.

Assessment of musculoskeletal disorders

Musculoskeletal symptoms were assessed using the Standardized Nordic Musculoskeletal Questionnaire (NMQ), a widely used and validated tool for epidemiological studies of musculoskeletal disorders [11]. The questionnaire evaluates the presence of symptoms in different body regions over specified time periods. The NMQ is available in the public domain and was used for non-commercial academic research; therefore, no formal permission was required. PRMDs were defined according to the criteria described by Christine Zaza [12] as pain, weakness, numbness, tingling, or other symptoms that interfere with the ability to play the instrument at the accustomed level. The questionnaire was translated into Bengali and contextually adapted for local use, with emphasis on maintaining conceptual equivalence of the original instrument.

Data analysis

Data were analyzed using descriptive statistics, as the study was designed to provide an exploratory overview rather than to test statistical associations.

Ethical considerations

Ethical approval was obtained from the Institutional Review Board of Bangabandhu Sheikh Mujib Medical University (BSMMU), now Bangladesh Medical University (BMU) (Approval No. BSMMU/2023/8052; Date: 06 June 2023). Written informed consent was obtained from all participants prior to data collection.

## Results

Participant characteristics and prevalence of PRMDs

A total of 116 professional tabla players were included in the study. Overall, 72% of participants reported experiencing PRMDs at some point in their lifetime, while 26% reported current symptoms at the time of assessment. Multiple anatomical site involvement was reported by 62% of participants with PRMDs. These findings are summarized in Table 1.

**Table 1:** Participant Characteristics and Prevalence of PRMDs (n = 116) PRMDs: Playing-related musculoskeletal disorders

Variable	n (%)
Total participants	116
Lifetime PRMD prevalence	84 (72)
Current PRMD prevalence	30 (26)
Multiple site involvement	72 (62)

Anatomical distribution of PRMDs

The most commonly affected anatomical sites were the wrist (14.5%) and shoulder (14.0%). Other frequently reported sites included the lower back (11.8%), fingers (10.8%), neck (8.1%), and elbow (7.0%) (Table 2). Overall, upper limb regions (including wrist, shoulder, elbow, and fingers) accounted for the majority of reported symptoms.

**Table 2:** Distribution of PRMDs by Anatomical Site (n = 116) Participants could report symptoms in more than one anatomical site. PRMDs: Playing-related musculoskeletal disorders

Anatomical Site	n (%)
Wrist	17 (14.5)
Shoulder	16 (14.0)
Elbow	8 (7.0)
Fingers	13 (10.8)
Lower back	14 (11.8)
Neck	9 (8.1)

Severity of symptoms

Among participants with current PRMDs, 38% reported mild symptoms, 45% reported moderate symptoms, and 17% reported severe symptoms.

Functional impact of PRMDs

PRMDs had a notable impact on function. More than half of the participants (58%) reported that their symptoms interfered with playing ability, while 42% experienced difficulty in performing daily activities.

Playing habits and PRMD trends

A descriptive trend suggested that participants with longer daily practice durations reported a higher prevalence of PRMDs. However, no inferential statistical analysis was performed to determine the significance of this association.

Preventive practices

Preventive strategies were infrequently practiced among participants. More than half (53%) reported not using any preventive measures. Among those who did, rest breaks (22%) were the most commonly reported, followed by warm-up exercises (15%) and ergonomic adjustments (10%).

## Discussion

This study demonstrates a high prevalence of PRMDs among Bangladeshi tabla players, with 72% reporting lifetime symptoms and 26% experiencing current complaints. These findings are consistent with recent international literature indicating that PRMDs remain one of the most prevalent occupational health problems among musicians, with reported prevalence rates ranging from 40% to 90% depending on instrument type and study methodology [13-16]. The relatively higher prevalence observed in this study may reflect instrument-specific biomechanical demands, limited ergonomic awareness, and lack of structured preventive programs in low- and middle-income settings.

The predominance of upper limb involvement, particularly the wrist and shoulder, is consistent with findings from contemporary studies of percussionists and other instrumental musicians, where the upper extremity is the most commonly affected region due to repetitive movements and sustained postural demands [3,4,9]. Similar patterns have also been reported in South Asian studies among tabla players, which highlight a high burden of upper limb symptoms, particularly involving the wrist [17,18].

Repetitive striking movements, high-frequency finger activity, and sustained non-neutral postures contribute to cumulative microtrauma affecting tendons, joints, and periarticular structures [16,19]. Electromyographic and kinematic studies have demonstrated that musicians performing repetitive upper limb tasks are exposed to significant mechanical load and muscle overuse, predisposing them to PRMDs [20-22]. In the context of tabla playing, the combination of rapid finger articulation and forceful impact on drum surfaces likely explains the high burden of wrist-related symptoms.

Lower back involvement observed in this study further highlights the role of posture in PRMD development. Prolonged unsupported sitting with forward trunk inclination has been identified as a key ergonomic risk factor among musicians, particularly in traditional performance settings [19]. Similar findings have been reported in studies of seated instrumentalists, where sustained spinal loading and inadequate postural support contribute to lumbar discomfort and chronic pain syndromes [23].

A notable finding of this study is the high proportion of participants reporting involvement of multiple anatomical sites (62%). This reflects the multifactorial and systemic nature of PRMDs, which often do not remain localized but evolve into widespread musculoskeletal dysfunction over time. Recent research emphasizes that PRMDs should be conceptualized not merely as localized injuries but as complex conditions influenced by biomechanical, psychosocial, and behavioral factors [24,25].

The functional impact of PRMDs observed in this study is clinically significant. More than half of the participants reported interference with playing ability, and a substantial proportion experienced limitations in daily activities. This aligns with growing evidence that PRMDs affect not only performance quality but also broader aspects of physical and psychosocial well-being, including fatigue, anxiety, and reduced career longevity [3,4,24,26]. These findings reinforce the importance of early identification and intervention to prevent progression and long-term disability.

The observed association between longer daily practice duration and higher prevalence of PRMDs, although descriptive, is consistent with established evidence identifying excessive practice load as a major risk factor. Studies have shown that prolonged practice without adequate rest leads to cumulative tissue strain, impaired recovery, and increased injury risk among musicians [16,19,21]. Load management principles, commonly applied in sports medicine, are increasingly recognized as relevant to performing artists and may help reduce PRMD burden.

One of the most concerning findings of this study is the limited use of preventive strategies. More than half of the participants reported not adopting any preventive measures, while only a minority engaged in warm-up exercises or ergonomic adjustments. This highlights a broader gap in awareness and access to musician-specific health education. Emerging interventional evidence suggests that structured exercise programs, posture training, and ergonomic modifications can significantly reduce the incidence and severity of PRMDs. Recent systematic and interventional studies have demonstrated that therapeutic exercise programs produce significant reductions in pain intensity among instrumental musicians, while combined approaches integrating education, exercise, and ergonomic strategies further improve outcomes and reduce symptom burden [27,28]. Additionally, targeted warm-up interventions have shown potential in decreasing pain intensity and interference, reinforcing the role of preventive conditioning in this population [29]. Despite this, such interventions remain underutilized, particularly in low-resource settings.

From a rehabilitation perspective, these findings highlight the critical role of physiatrists and multidisciplinary teams in addressing PRMDs. Contemporary models of performing arts medicine emphasize integrated care involving physical therapy, ergonomic assessment, behavioral modification, and load management [30]. Incorporating rehabilitation services into musician health programs may facilitate early detection, optimize functional outcomes, and support career sustainability.

The study has several limitations that should be acknowledged. The use of purposive sampling and recruitment from selected settings may limit the generalizability of the findings to the broader community of musicians, and the inclusion of predominantly male participants restricts applicability to female musicians that may limit the applicability of the findings to musicians in other countries. Additionally, the achieved sample size (n = 116) was lower than the calculated requirement (n = 174), which may reduce statistical power and limit the representativeness of the findings. The reliance on self-reported questionnaire-based data introduces the possibility of recall bias and reporting bias, which may affect the accuracy of symptom prevalence and severity. Although the standardized NMQ is a validated tool, the adapted and Bengali-translated version used in this study was not formally revalidated, which may affect measurement accuracy and internal consistency. The absence of physical examination and objective diagnostic assessment limits the ability to clinically confirm musculoskeletal disorders and may reduce the clinical reliability of the findings. In addition, important factors such as posture, playing technique, physical fitness, psychosocial influences, and ergonomic setup were not assessed in depth, which may limit a comprehensive understanding of the determinants of PRMDs. The use of descriptive statistics without inferential analysis limits the ability to assess statistical associations between variables; therefore, observed trends should be interpreted cautiously and cannot be considered confirmatory. Similarly, although preventive practices were reported, their association with PRMD outcomes was not analyzed, and their effectiveness cannot be determined from this study. The cross-sectional design precludes the establishment of cause-and-effect relationships; therefore, observed associations, including those related to practice duration, should be interpreted cautiously and not as evidence of causality. Future studies incorporating clinical evaluation and longitudinal designs are warranted.

## Conclusions

PRMDs appear to be highly prevalent among Bangladeshi tabla players, predominantly affecting the upper limb and impacting functional performance. However, given the methodological limitations of this study, these findings should be interpreted with caution. The results provide preliminary insights that may inform future research. Further studies using robust sampling strategies, analytical designs, and objective clinical assessments are required to guide evidence-based clinical and preventive interventions.
